# COL1A1, PRPF40A, and UCP2 correlate with hypoxia markers in non-small cell lung cancer

**DOI:** 10.1007/s00432-017-2381-y

**Published:** 2017-03-03

**Authors:** Urszula Oleksiewicz, Triantafillos Liloglou, Kalliopi-Maria Tasopoulou, Nikoleta Daskoulidou, John R. Gosney, John K. Field, George Xinarianos

**Affiliations:** 1 Roy Castle Lung Cancer Research Programme, University of Liverpool Cancer Research Centre, 200 London Rd, Liverpool, L3 9TA UK; 2 Department of Pathology, University of Liverpool, Duncan Building, Daulby Street, Liverpool, L69 3GA UK; 3 Department of Cancer Immunology, Poznan University of Medical Sciences, 15 Garbary, 61-866 Poznan, Poland; 4 Department of Surgery, General Hospital of Didymoteicho, 152 25th Maiou street, 68300 Didymoteicho, Greece; 5 Faculty of Health and Medical Sciences, University of Surrey, AY Building, Guildford, Surrey GU2 7 XH UK

**Keywords:** COL1A1, PRPF40A, UCP2, Hypoxia, Non-small cell lung cancer

## Abstract

**Purpose:**

Collagen 1A1 (COL1A1), RNA-binding and pre-mRNA Processing Factor (PRPF40A), and Uncoupling Protein 2 (UCP2) were identified as downstream effectors of cytoglobin (CYGB), which was shown implicated in tumour biology. Although these three genes have been previously associated with cancer, little is known about their status in lung malignancies.

**Methods:**

Hereby, we investigated the expression and promoter methylation of COL1A1, PRPF40A, and UCP2 in 156 non-small cell lung cancer (NSCLC) and adjacent normal tissues.

**Results:**

We demonstrate that COL1A1 and PRPF40A mRNAs are significantly overexpressed in NSCLC (*p* < 1 × 10^−4^), while UCP2 exhibits a trend of upregulation (*p* = 0.066). Only COL1A1 promoter revealed hypermethylation in NSCLCs (36%), which was particularly evident in squamous cell carcinomas (*p* = 0.024) and in the tumours with moderate-to-good differentiation (*p* = 0.01). Transcript level of COL1A1, as well as PRPF40A and UCP2, exhibited striking association (*p* ≤ 0.001) with the expression of hypoxia markers. In addition, we demonstrate in lung cancer cell lines exposed to hypoxia or oxidative stress that COL1A1 transcription significantly responds to oxygen depletion, while other genes showed the modest upregulation in stress conditions.

**Conclusion:**

In conclusion, our data revealed that COL1A1, UCP2, and PRPF40A are novel players implicated in the complex network of hypoxia response in NSCLC.

## Introduction

Lung cancer is among the most prevalent neoplastic diseases accounting for the highest mortality in both genders worldwide (Boisvert et al. 2010). Due to typically asymptomatic early stage and late diagnosis, lung tumours are difficult to treat as the therapeutic options become limited. The reduction of lung cancer mortality has been set as a major priority in the UK through the development of more efficient early diagnosis and intervention tools, both of which require better understanding of the molecular biology of the disease (Lung et al. 2000).

Our previous studies suggested cytoglobin (CYGB) as a putative tumour suppressor gene in lung (Xinarianos et al. 2006) and oesophageal (McRonald et al. 2006) tumours and successive in vitro and in vivo studies provided further evidence in support of this hypothesis (Oleksiewicz et al. 2013; Shivapurkar et al. 2008; Thuy le et al. 2011). Nevertheless, CYGB was also associated with hypoxia and cancer aggressiveness (Fang et al. 2011; Oleksiewicz et al. 2013). Shivapurkar and co-workers identified three downstream effector genes of CYGB, which were downregulated in CYGB-overexpressing lung and breast cancer cell lines (Shivapurkar et al. 2008). These genes were: collagen 1A1 (COL1A1), RNA-binding and pre-mRNA Processing Factor (PRPF40A) and Uncoupling Protein 2 (UCP2). Interestingly, COL1A1, PRPF40A, and UCP2 expression was linked to the metabolic pathways implicated in hypoxia and oxidative stress (Baffy 2010; Falanga et al. 2002; García-Trevijano et al. 1999; Papaiahgari et al. 2007), events associated with more aggressive and therapy-resistant tumours (Harris 2002; Landriscina et al. 2009).

COL1A1 is an extracellular matrix protein, whose overexpression was linked to breast (Jansen et al. 2005), gastric (Yasui et al. 2005), and colorectal tumours (Steinman 2012). UCP2 is a mitochondrial membrane protein transporting protons from the intermembrane space to mitochondrial matrix (i.e., uncoupling). Upregulation of UCP2 was reported in colon cancer (Horimoto et al. 2004), hepatocellular cancer, and cholangiocarcinoma (Baffy 2010). Finally, PRPF40A participates in assembly of splicing machinery onto the pre-mRNAs (Lin et al. 2004). Little is known about the involvement of PRPF40A in cancer. However, its upregulation was reported in pancreatic cancer (Thakur et al. 2008) and in the cells harbouring various p53 cancer-related mutations (Randolph et al. 1999).

In previous microarray analyses, COL1A1 (Ramaswamy et al. 2003) and UCP2 (Ayyasamy et al. 2011) were found upregulated in various malignant tissues, including lung cancer. However, no single-gene validation of these results in lung cancer has been presented so far. Thus, we aimed to explore UCP2, PRPF40A and COL1A1 mRNA expression, and promoter methylation to determine their molecular profile in NSCLC and their association to clinicopathological parameters and hypoxia markers.

## Materials and methods

### Tissue collection

NSCLC and adjacent normal tissues were collected from 156 patients operated between 1995 and 2005 at the Liverpool Heart and Chest Hospital, UK. All patients submitted informed consent, and the study was approved by the local ethics committee. The patient group [described in (Oleksiewicz et al. 2011)] represents typical cohort with resectable lung tumour as decided following standard diagnostic procedures (Table 1). All tumour samples come from the patients that did not receive any prior chemo- or radio-therapy. The patient cohort consisted of 99 males and 57 females, with the mean age 65.7 (range 40–87). This set contained two most frequent histological subtypes: squamous cell lung cancer (SqCLC) (*N* = 86) and adenocarcinoma (*N* = 70). With regard to the pathological stage, tumours were split into the following groups: T1 (*N* = 11), T2 (*N* = 125), T3 (*N* = 15), and T4 (*N* = 5), while according to differentiation status, the group breakdown was: poor (*N* = 48) and moderate/good (*N* = 107). Nodal metastasis occurred in 75 cases, while 80 patients had metastasis-free nodes. The follow-up data included 12 alive and 57 dead patients. The tumour samples were snap frozen and grossly micro-dissected to ascertain more than 80% tumour tissue. The normal adjacent tissue was resected at least 5 cm from the tumour mass. All the methylation and expression values were included in the analysis, even in the absence of normal, methylation, or expression counterpart data for the given patient. Overall, we utilised RNA from 128 normal and 146 NSCLC tissues and DNA from 26 normal and 91 NSCLC tissues.

**Table 1:** Clinicopathological data available for the analysed NSCLC population

Clinical parameters	Data for all patients (*N* = 156)	Data for methylation analysis (*N* = 91)	Data for pairwise methylation analysis (*N* = 26)
Histology			
SqCCL	86 (55%)	56 (62%)	15 (58%)
AdenoCa	70 (45%)	35 (38%)	11 (42%)
Differentiation			
Poor	48 (31%)	26 (29%)	8 (31%)
Moderate to good	107 (69%)	65 (71%)	18 (69%)
Tumour stage			
T1	11 (7%)	4 (4%)	2 (8%)
T2	125 (80%)	74 (81%)	21 (81%)
T3	15 (10%)	9 (10%)	2 (8%)
T4	5 (3%)	4 (4%)	1 (4%)
Nodal status			
Negative	75 (48%)	44 (49%)	17 (65%)
Positive	80 (52%)	46 (51%)	9 (35%)
Gender			
Male	99 (63%)	62 (68%)	19 (73%)
Female	57 (37%)	29 (32%)	7 (27%)
Follow-up			
Alive	12 (17%)	9 (18%)	5 (28%)
Dead	57 (83%)	40 (82%)	19 (73%)
Age			
Mean age (range)	66 (40–87)	66 (40–87)	64 (40–87)

### Cell lines

Adenocarcinoma (H358) and SqCLC (CALU1) lung cancer cell lines (ATCC) were maintained at 37 °C/5% CO_2_ in DMEM/F-12/Glutamax™-I (Invitrogen) with 10% FBS. For hypoxic studies, the cells were cultured at 1% O_2_ for 48h, while oxidative stress was achieved with 300 µM H_2_O_2_ treatment for 24 h. H_2_O_2_ concentration was chosen as an IC50 of CALU1 and H358 according to our survival curve.

### Promoter methylation

DNA extraction, bisulfite conversion, and pyrosequencing analyses were performed as described (Oleksiewicz et al. 2011). PCR reactions were performed with HotStarTaq^®^Plus Mastermix (Qiagen) according to manufacturer’s protocol with 85 ng bisulfite-converted DNA and 0.16 µM primers. The following PCR primers (MWG Operon) were used: COL1A1_F GGAGAGAAGGTAAATGGAAG, COL1A1_R-biotinylated AACCTAACCCCAACCCTA, PRPF40A_F GAGTAGAGAATAGAGAGGATTTG, PRPF40A_R-biotinylated TAATCAAAACCCAAAAAAC, UCP2_F-biotinylated GTTTTGGGATTGATTGTT, and UCP2_R CAAAACTAAAACCAAACTCAC. For pyrosequencing reaction, 0.33 µM sequencing primer was used: COL1A1_S (GAGAAGGTAAATGGAAGA), PRPF40A_S (ATAGAGAGGATTTGGA), and UCP2_S (AAACTAAAACCAAACTC).

### Gene expression

RNA isolation, reverse transcription, qPCR, and data analysis were previously described (Oleksiewicz et al. 2011). The following primer/probe mixes were used in the qPCRs: COL1A1 (Hs01076780_g1), PRPF40A (Hs00215465_m1), and UCP2 (Hs01075225_m1) (Life Technologies).

### GSH-Glow™ glutathione assay

Cellular level of glutathione was measured with the GSH-Glow™ glutathione assay (Promega) on Genios plate reader (Tecan) according to manufacturer’s instructions. The luminescence values were normalised to the total protein level as assessed with the DC Biorad Assay (Biorad).

### Statistical analysis

Non-parametric tests were used for statistical analyses (PASW Statistics 18.0, SPSS) as 1-sample Kolmogorov–Smirnov test showed skewed distribution of continuous variables. Pairwise comparisons between normal and NSCLC samples were performed with Wilcoxon test. The differences for continuous variables among independent cohorts within clinical parameters were determined with the Kruskal–Wallis and Mann–Whitney tests. Bivariate correlation was probed with Spearman’s test. Bonferroni correction was applied to adjust for multiple comparisons. Patients’ survival was calculated with the log rank test.

## Results

### Gene expression analysis of COL1A1, PRPF40A, and UCP2 in NSCLC

**Fig. 1:**
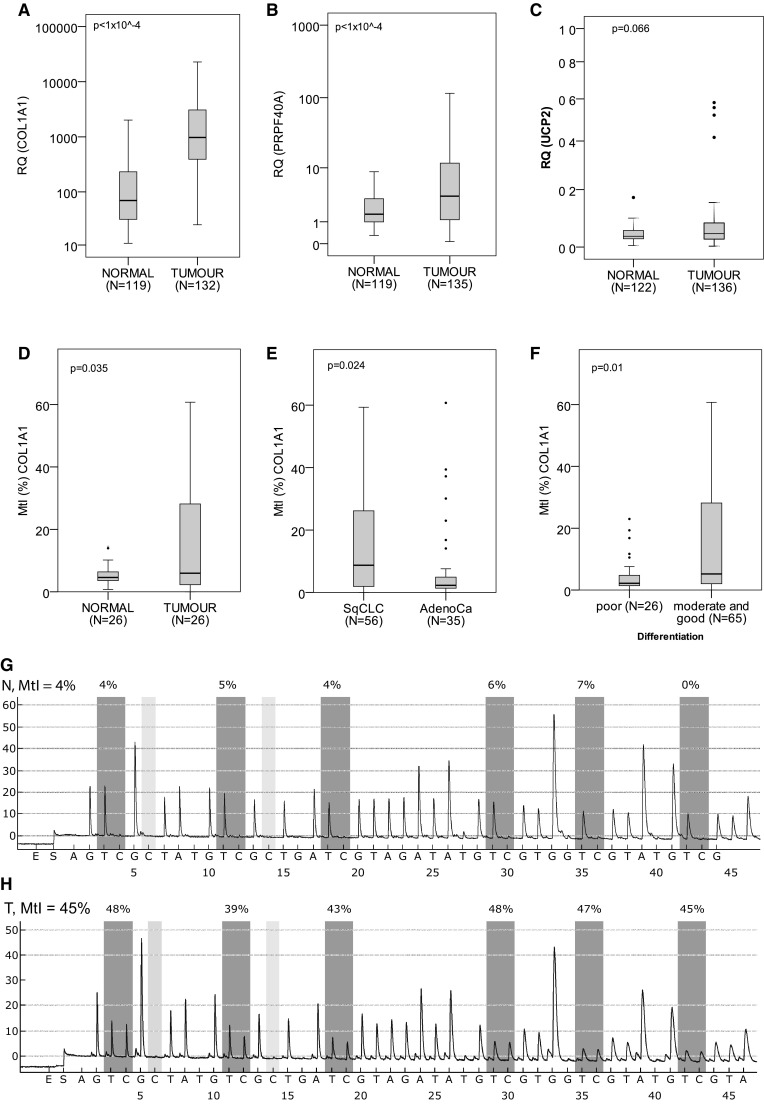
Boxplot representation of **a** COL1A1, **b** PRPF40A, and **c** UCP2 mRNA levels in NSCLC and normal tissues. **d** *COL1A1* promoter methylation profile in NSCLC and normal samples, **e** in adenocarcinomas and SqCLCs, and **f** in the tumours with poor and moderate-to-good differentiation. A number of cases analysed with the Mann–Whitney (**a**–**c, e, f**) or Wilcoxon (**d**) test and corresponding *p* values are provided. Representative pyrograms of COL1A1 promoter for normal (**g**) and NSCLC (**h**) samples. *X*-axis indicates nucleotide dispensation order, while *Y*-axis luminescence intensity for each incorporated nucleotide. *Boxes* above the *graphs* show the percentage of methylated cytosines for each CpG site

Our comparative qPCR analysis showed abundant COL1A1 overexpression in tumours (median RQ = 975.4, IQR 389.9–3172.7, *N* = 132) when compared to adjacent normal tissues (69.4, 29.8–235.6, *N* = 119, Mann–Whitney test, *p* < 1 × 10^−4^) (Fig. 1a). Similarly, PRPF40A was overexpressed in NSCLC samples (RQ = 3.49, 1.13–11.84, *N* = 135 vs 1.53, 0.98–3.16, *N* = 119, Mann–Whitney test, *p* < 1 × 10^−4^) (Fig. 1b). In the case of UCP2, there was only a trend of higher expression observed in lung tumours (RQ = 0.043, 0.025–0.08, *N* = 136 vs 0.034, 0.026–0.055, *N* = 122, Mann–Whitney test, *p* = 0.066) (Fig. 1c). UCP2 mRNA expression was higher in lung adenocarcinomas (RQ = 0.053, 0.03–0.087, *N* = 61) than in SqCLCs (RQ = 0.035, 0.019–0.072, *N* = 75, Mann–Whitney test, *p* = 0.015). Apart from that no other associations were observed between COL1A1, PRPF40A or UCP2 mRNA expression and patients’ gender, age, survival, smoking history, TNM classification, and tumour histology and differentiation.

### Promoter methylation analysis of COL1A1, PRPF40A, and UCP2 in NSCLC

For the promoter methylation analysis, we set the hypermethylation threshold according to the previously described method (Normal reference range = mean MtI + 2 × standard deviation of the normal samples) (Oleksiewicz et al. 2011) at MtI = 19.9% for UCP2 and MtI = 13.0% for COL1A1. PRPF40A promoter was unmethylated in all samples tested. UCP2 promoter hypermethylation was observed only in 5/91 NSCLC samples and none (0/26) of the normal tissues. We noted elevated COL1A1 promoter methylation in 33/91 NSCLCs (Fig. 1D) and in 2/26 normal samples. Although the difference in methylation between grouped normal and tumour samples was insignificant, pairwise comparison showed significant hypermethylation of COL1A1 promoter in NSCLC (*p* = 0.035, Wilcoxon test, *N* = 26 pairs). The samples selected for the latter analysis featured clinicopathological profile representative of all tumour samples utilised in this study (Table 1), so that the pairwise comparison was not confounded by the clinical factors. Furthermore, higher methylation was observed in SqCLCs (median MtI = 8.72%, IQR 1.95–26.18, *N* = 56) than in adenocarcinomas (2.28, 1.36–5.5, *N* = 35, Mann–Whitney test, *p* = 0.024, Fig. 1e), as well as in the moderately and well-differentiated tumours (MtI = 5.19%, IQR 1.99–28.21, *N* = 65) in comparison with poorly differentiated NSCLCs (2.18, 1.39–5.45, *N* = 26, Mann–Whitney test, *p* = 0.01, Fig. 1f). Apart from that observation COL1A1 methylation did not correlate with patients’ gender, age, survival, smoking history, and TNM classification. Moreover, methylation was not associated with the mRNA expression values, even when potentially confounding factors (histology and differentiation) were taken into account.

### COL1A1, PRPF40A, and UCP2 expression correlates with hypoxia markers

**Fig. 2:**
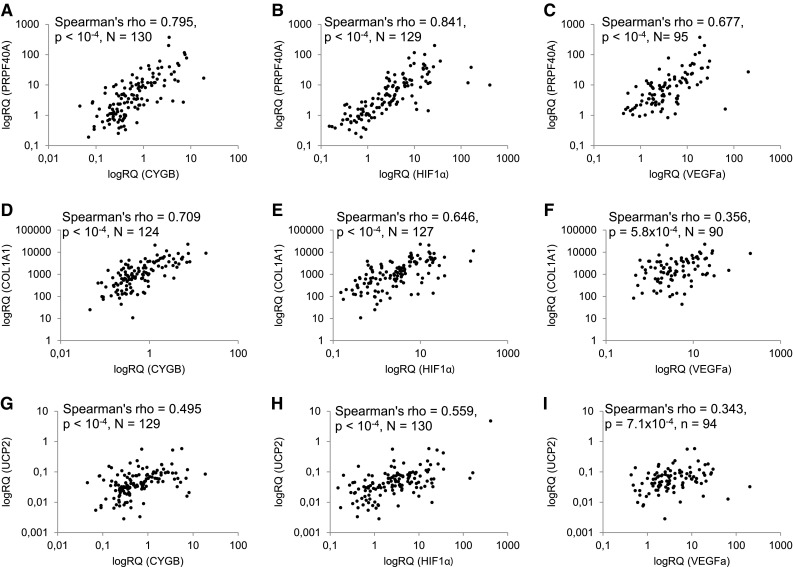
Scatterplots representing correlations between mRNA expression of PRPF40A (**a**–**c**), COL1A1 (**d**–**f**), UCP2 (**g**–**i**), and hypoxia markers: CYGB (**a, d, g**), HIF1α (**b, e, h**), or VEGFa (**c, f, i**) in NSCLC resected tissues. The *p* and rho values and numbers of paired samples included in the Spearman’s correlation test are indicated on each graph

Next, we evaluated interrelationships between COL1A1, PRPF40A, UCP2, and hypoxia markers (CYGB, Hypoxia-Inducible Factor 1α—HIF1α and Vascular Endothelial Growth Factor—VEGFa), whose expression profiles were previously reported (Oleksiewicz et al. 2011). PRPF40A exhibited the strongest hypoxia association pattern among all genes under investigation (Table 2; Fig. 2a–c). Its mRNA expression level correlated with CYGB (*ρ* = 0.795, *p* < 1 × 10^−4^, *N* = 130), HIF1α (*ρ* = 0.841, *p* < 1 × 10^−4^, *N* = 129), and VEGFa (*ρ* = 0.677, *p* < 1 × 10^−4^, *N* = 95). The hypoxia dependence was observed as well in the case of COL1A1 (Fig. 2d–f), whose expression was associated with CYGB (*ρ* = 0.709, *p* < 1 × 10^−4^, *N* = 124), HIF1α (*ρ* = 0.646, *p* < 1 × 10^−4^, *N* = 127) and, to a lesser extent, with VEGFa (*ρ* = 0.356, *p* = 5.8 × 10^−4^, *N* = 90). Similar relationships were seen in the case of UCP2 (UCP2 vs CYGB: *ρ* = 0.495, *p* < 1 × 10^−4^, *N* = 129, UCP2 vs HIF1α: *ρ* = 0.559, *p* < 1 × 10^−4^, *N* = 130 and vs VEGFa: *ρ* = 0.343, *p* = 7.1 × 10^−4^, *N* = 94, Fig. 2g–i). The expression profiles of COL1A1, PRPF40A, and UCP2 genes correlated with each other. The strongest positive association was observed between PRPF40A and COL1A1 (*ρ* = 0.612, *p* < 1 × 10^−4^, *N* = 129), PRPF40A and UCP2 (*ρ* = 0.596, *p* < 1 × 10^−4^, *N* = 132), while the weakest between COL1A1 and UCP2 (*ρ* = 0.353, *p* < 1 × 10^−4^, *N* = 128).

**Table 2:** Association between mRNA expression of COL1A1, PRPF40A, UCP2, CYGB, HIF1α, and VEGFa assessed with Spearman’s test

	PRPF40A	UCP2	CYGB	HIF1α	VEGFa
COL1A1	Rho = 0.612 *p* < 1 × 10^−4^ *N* = 129	Rho = 0.353 *p* < 1 × 10^−4^ *N* = 128	Rho = 0.709 *p* < 1 × 10^−4^ *N* = 124	Rho = 0.646 *p* < 1 × 10^−4^ *N* = 127	Rho = 0.356 *p* = 5.8 × 10^−4^ *N* = 90
PRPF40A		Rho = 0.596 *p* < 1 × 10^−4^ *N* = 132	Rho = 0.795 *p* < 1 × 10^−4^ *N* = 130	Rho = 0.841 *p* < 1 × 10^−4^ *N* = 129	Rho = 0.677 *p* < 1 × 10^−4^ *N* = 95
UCP2			Rho = 0.495 *p* < 1 × 10^−4^ *N* = 129	Rho = 0.559 *p* < 1 × 10^−4^ *N* = 130	Rho = 0.343 *p* = 7.1 × 10^−4^ *N* = 94

Rho values (correlation coefficient), *p* values, and the number of samples for each analysis are provided

### COL1A1, PRPF40A, and UCP2 expression under stress conditions in vitro

**Fig. 3:**
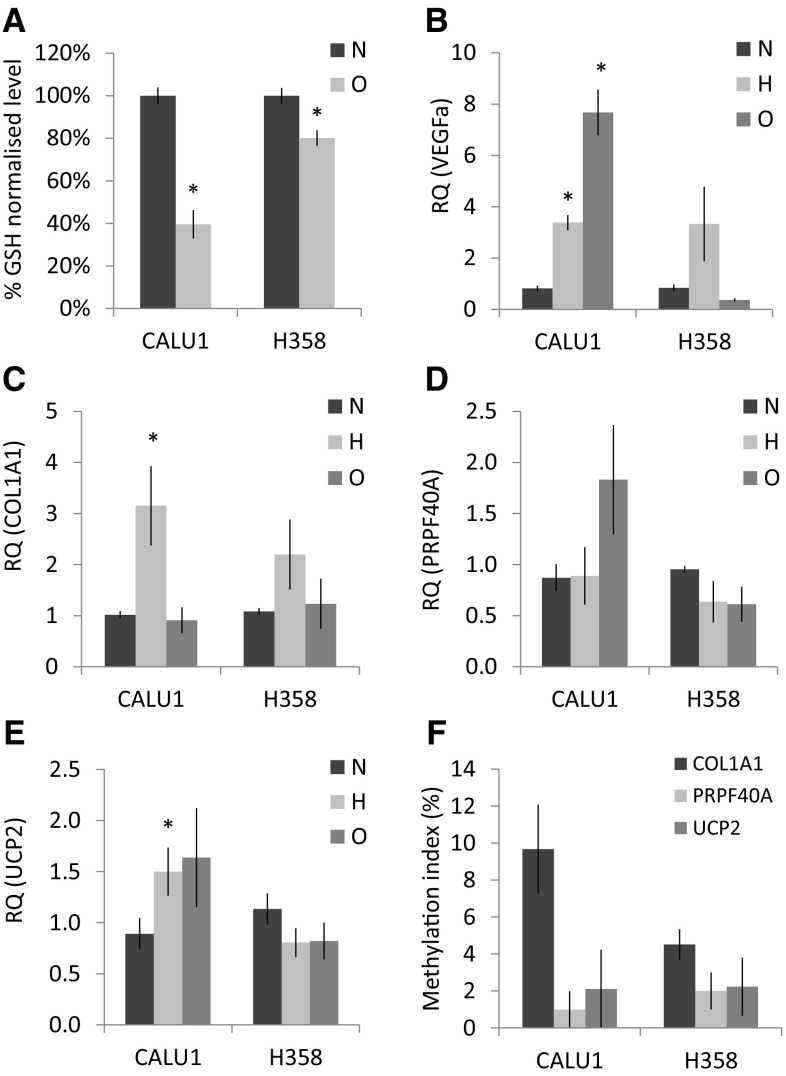
Glutathione (GSH) level normalised to total protein content **a** in CALU1 and H358 cell lines after 24h incubation with 300 µM H_2_O_2_ (*N* = 6). Relative mRNA expression of **b** VEGFa, **c** COL1A1, **d** PRPF40A, and **e** UCP2 in CALU1 and H358 cell lines after 48h incubation at 1% O_2_ **(h)** or 24 h incubation with 300 µM H_2_O_2_ (**o**). *Bars* represent mean (*N* = 4) RQ values (±SEM) calibrated against untreated (*N*) control cells. *p < 0.05 (Mann–Whitney test). Pyrosequencing analysis of CpG sites within COL1A1 (*N* = 6 CpG sites), PRPF40A (*N* = 7), and UCP2 (*N* = 5) promoters in CALU1 and H358 (**f**). The *bars* represent mean methylation index (MtI ± SEM of CpG sites)

Hypoxia response is activated not only with oxygen depletion, but also with nutrient deficiency, oxidative stress, and other signalling pathways. Therefore, we wanted to assess whether COL1A1, PRPF40A, and UCP2 might be regulated by hypoxia and/or oxidative stress in vitro. Cellular response to oxidative and hypoxic stress was confirmed by testing glutathione content (Fig. 3a) and the mRNA expression of VEGFa (Fig. 3b), respectively. COL1A1 became upregulated in hypoxic conditions in both cell lines; however, this was significant only in CALU1 (RQ = 3.15 ± 0.8 vs 1.0 ± 0.07 in normoxic cells, *p* = 0.02, Mann–Whitney), but not in H358 (RQ = 2.2 ± 0.7 vs 1.1 ± 0.06, *p* = 0.074). PRPF40A and UCP2 expression little changed at 1% O_2_, as only CALU1 exhibited modest upregulation of UCP2 (RQ = 1.5 ± 0.24 vs 0.9 ± 0.15, *p* = 0.04, Mann–Whitney). Similarly, oxidative stress did not evoke significant changes in the expression of COL1A1, UCP2, and PRPF40A genes. This lack of responsiveness to stress conditions was not caused by CpG methylation, as the promoters of COL1A1, UCP2, and PRPF40A showed low methylation level (MtI < 10%) in both cell lines (Fig. 3f).

## Discussion

Although UCP2, PRPF40A, and COL1A1 upregulation has been previously observed in malignant tissues (Ayyasamy et al. 2011; Ramaswamy et al. 2003; Thakur et al. 2008) and associated with response to hypoxia and oxidative stress (Baffy 2010; Falanga et al. 2002; García-Trevijano et al. 1999; Papaiahgari et al. 2007), little is known about their status in lung cancer. The data presented hereby demonstrate a trend of overexpression of UCP2 and significant overexpression of COL1A1 and PRPF40A in NSCLCs compared to adjacent normal tissues. Moreover, expression of all three genes correlated with that of hypoxia markers: CYGB, HIF1α, and VEGFa. Furthermore, we observed that PRPF40A promoter remained unmethylated, while UCP2 promoter was sporadically methylated in few cancer samples. Interestingly, we demonstrated a trend of increased methylation within COL1A1 promoter in a subset of NSCLCs; however, it did not associated with COL1A1 expression. This might be due to insufficient sample number taken into analysis, existence of another promoter controlling COL1A1 expression or presence of a potent factor that affects COL1A1 mRNA content more profoundly than DNA methylation, e.g., tumour hypoxia. Indeed, COL1A1 expression strongly correlates with hypoxia markers in tissues and becomes upregulated as well in low oxygen tension in our cell line model. Thus, the effect of hypoxia may be masking the methylation events observed in the subset of the NSCLC tissues. Surprisingly, our data indicate positive correlation of COL1A1, UCP2, and PRPF40A with CYGB expression, which has been previously shown to be inverse in in vitro experiments (Shivapurkar et al. 2008). In the data published by Shivapurkar et al., transient CYGB overexpression resulted in >twofold downregulation of the three genes; however, this effect was less pronounced and reproducible in the cells with stable CYGB overexpression. While in vivo data not always recapitulate in vitro observations, prolonged tumour hypoxia identified in our tissue set may additionally modify the interdependence of CYGB and its proposed downstream effectors. For example, in tumour microenvironment, CYGB may undergo functional alterations that affect its properties as the regulator of downstream genes.

Despite the clustering of the three genes with hypoxia markers in tissues, in vitro hypoxia had differential effect on their expression. The statistical correlation between various genes might reflect similar, but unrelated expression patterns. These patterns could manifest the specificity of pathologic transcriptome rather than the activation of a particular signalling pathway. From this point of view, lack of responsiveness in the cells incubated at low O_2_ level may indicate independence of PRPF40A and UCP2 from hypoxia in NSCLCs. It is possible as well that in the lung cancer setting, UCP2 and PRPF40A act upstream of hypoxia pathways, thus inducing expression of HIF1α independently of the O_2_ tension. Alternatively, the expression of PRPF40A and UCP2 may require additional stimulators, which are present in tumour microenvironment in vivo, but absent in vitro (e.g., more acute O_2_ deficit, cytokine release, and interactions with extracellular stroma) (Creighton et al. 2005). Falanga and colleagues, for instance, reported that in dermal fibroblasts, hypoxia augments the transcription and extracellular deposition of COL1A1 via TGF-b pathway (Falanga et al. 2002). Although the three genes have been previously shown upregulated upon hypoxic stimulus at the mRNA and/or protein level (Deng et al. 2012; Falanga et al. 2002; Lai et al. 2016), observations in other experimental settings suggest their hypoxia-driven downregulation (Duval et al. 2016; Perry et al. 2013; Wang et al. 2016). Altogether these data indicate that the responsiveness to low O_2_ level by COL1A1, UCP2, and PRPF40A is dependent on cellular context.

While UCP2 overexpression might contribute to Warburg effect and promote tumourigenic phenotype (Ayyasamy et al. 2011), the implications of deregulated expression patterns of PRPF40A and COL1A1 remain to be elucidated. It is likely, however, that these genes may assist in the adaptive responses to oxidative stress and hypoxia/reoxygenation events, thus promoting tumour aggressiveness, metastasis, and treatment resistance. As these issues are pertinent to cancer patient management, it would of interest to investigate this hypothesis in the future.
